# Insulin potentiates mechanical responses in small dorsal root ganglion neurons by increasing the sensitization of TRPV4 channels

**DOI:** 10.1152/ajpcell.00255.2025

**Published:** 2025-05-09

**Authors:** Ayumi Fukazawa, Amane Hori, Juan Estrada, Han-Kyul Kim, Norio Hotta, Gary A. Iwamoto, Scott A. Smith, Wanpen Vongpatanasin, Masaki Mizuno

**Affiliations:** 1 Department of Applied Clinical Research, University of Texas Southwestern Medical Center, Dallas, Texas, United States;; 2 Japan Society for the Promotion of Science, Tokyo, Japan;; 3 College of Life and Health Sciences, Chubu University, Kasugai, Japan;; 4 Department of Internal Medicine, University of Texas Southwestern Medical Center, Dallas, Texas, United States;; 5 Department of Surgery, University of Texas Southwestern Medical Center, Dallas, Texas, United States

**Keywords:** dorsal root ganglion, insulin, insulin receptor signaling, mechanical sensitization, transient receptor potential vanilloid 4

## Abstract

We have previously reported that insulin potentiates the response to mechanical stimuli in small dorsal root ganglion (DRG) neurons. However, the mechanisms underlying the insulin-induced potentiated responsiveness to mechanical stimulation in sensory neurons remain unclear. Transient receptor potential vanilloid 4 (TRPV4) is expressed as a mechanosensitive channel in DRG neurons and is activated by mechanical stimuli. We therefore hypothesized that insulin augments the response to mechanical stimulation in small DRG neurons by enhancing sensitization of TRPV4 channels. Colocalization of TRPV4, insulin receptor (IR), and the C-fiber marker peripherin in small DRG neurons was evaluated by immunofluorescence, demonstrating that 53 ± 10% of TRPV4-positive small DRG neurons were colocalized with IR and peripherin. In in vitro whole cell patch clamp recordings from cultured DRG neurons, mechanically activated currents were significantly increased 5 min after the application of insulin (*P* = 0.0137) and such augmentation was suppressed by TRPV4 antagonist HC067047. We further examined the impact of insulin on the expression of the IR signaling pathway proteins in cultured DRG neurons using western blotting. Akt was significantly increased in cultured DRG neurons incubated with insulin (phospho-Akt: *P* = 0.0007, phospho/total Akt ratio: *P* = 0.0183). Furthermore, blocking IR signaling kinases, phosphoinositide 3-kinase (PI3K), and PKC suppressed the insulin-induced augmentation in TRPV4 agonist-induced currents (PI3K: *P* = 0.0074, PKC: *P* = 0.0028). Collectively, our results suggest that insulin-induced potentiation of mechanical response in small DRG neurons occurs through enhanced sensitization of TRPV4 channels.

## NEW & NOTEWORTHY

We investigated whether insulin potentiates the sensitization of TRPV4 channels to mechanical stimulation in DRG neurons. Insulin-induced enhancement of mechanical response was suppressed by blocking TRPV4 channels. Furthermore, blockade of insulin receptor signaling pathways, PI3K and PKC, inhibited insulin-induced sensitization of TRPV4 channels. Our results provide evidence that insulin-induced potentiation of mechanical sensation in small DRG neurons is mediated through enhancing the sensitization of TRPV4 channels via the insulin receptor signaling pathway.

## INTRODUCTION

Insulin is not only known as the hormone responsible for maintaining glucose homeostasis (1) but it also modulates the function of some ion channels (2, 3). We have previously shown that insulin potentiates the responsiveness of dorsal root ganglia (DRG) neurons and potentially sensitizes thin fiber muscle afferents to mechanical stimuli (4). However, it is fully unknown which mechanosensitive channels can be sensitized by the insulin signaling pathway.

Transient receptor potential vanilloid 4 (TRPV4) is well known as a mechanosensitive channel and plays a crucial role in mechanical response in endothelial cells (5), smooth muscle cells (6), and DRG neurons. Our recent study suggests that antagonism of TRPV4 channels significantly lowered the sensitivity to mechanical stimuli in small DRG neurons (7). That being said, it remains unclear whether insulin potentiates the activity of TRPV4 channels. Increasing evidence suggests that TRPV4 can be sensitized by protein kinase C (PKC), which is indirectly activated by insulin through common pathways involving phosphoinositide 3-kinase (PI3K) (8–10). In addition, it has been reported that insulin receptors (IRs) (11, 12) and TRPV4 (9, 13) are coexpressed in DRG neurons. Thus, it is hypothesized that the insulin signaling pathway involving PI3K and PKC potentiates mechanical sensation in small DRG neurons by enhancing the sensitization of TRPV4 channels.

The present study therefore aimed to determine the extent to which *1*) TRPV4 and IR are colocalized in C-fiber DRG neurons, *2*) inhibition of TRPV4 channels attenuates insulin-induced augmentation of mechanically activated (MA) inward currents, *3*) insulin application increases the expression of proteins involved in insulin signaling pathways such as PI3K, Akt, and PKC, in cultured DRG neurons, and *4*) inhibition of the insulin signaling pathway attenuates the insulin-induced enhancement of TRPV4 agonist-induced inward currents.

## MATERIALS AND METHODS

### Ethical Approval

All studies were performed in accordance with the US Department of Health and Human Services NIH Guide for the Care and Use of Laboratory Animals. All experimental procedures were approved by the Institutional Animal Care and Use Committee of the University of Texas Southwestern Medical Center (no. 2019–102849).

### Animals

Thirty-three male Sprague-Dawley (SD) rats [body weight (BW): 312 ± 41 g, 7–12 wk of age] (Inotiv, Indianapolis, IN) were used for immunofluorescence, whole cell patch clamp recording, and Western blotting. Animals had free access to food and water. The animals were kept one to four per cage under 12-h light-dark cycle in an air-conditioned room (22–24°C) until required for experiments.

### Immunofluorescence

Rats were given Euthasol anesthesia [390 mg/mL pentobarbital sodium and 50 mg/mL phenytoin sodium, 0.1 mL/100 g body wt (0.28 mL–0.36 mL)]. The adequacy of anesthesia was verified by lack of a withdrawal response to tail pinch. Rats were then transcardially perfused with saline followed by 4% paraformaldehyde (PFA) for tissue fixation. The L4–6 DRGs were harvested and submerged in 4% PFA overnight. The DRGs were dehydrated sequentially in 10% and 20% sucrose and afterward sectioned at 10 μm using a cryostat. Coimmunostaining of TRPV4, IR, and the C-fiber marker peripherin was performed by incubation with guinea pig anti-TRPV4 (1:1,000, Cat. No. ACC-034-GP, RRID#AB_2876796; Alomone Labs, Jerusalem, Israel), rabbit anti-IR (1:500, Cat. No. ab303492, RRID#AB_3083575; Abcam, Cambridge, MA), and anti-mouse peripherin (1: 250, Cat. No. ab254137, RRID# AB_3083576; Abcam) overnight at room temperature (RT), after blocking with 10% normal goat serum/phosphate-buffered saline (PBS) with 0.5% Triton X-100 for 1 h. The sections were rinsed in PBS and subsequently incubated with fluorescence-conjugated secondary antibodies [Alexa Fluor 555-conjugated goat anti-guinea pig IgG (1:500, Cat. No. A21435, RRID# AB_2535856; Invitrogen, Carlsbad, CA), Alexa Fluor 405-conjugated goat anti-rabbit IgG (1:500, Cat. No. A48254, RRID# AB_2890548; Invitrogen), and Alexa Fluor 488-conjugated goat anti-mouse IgG (1:500, Cat. No. A32723, RRID# AB_2633275; Invitrogen)] for 1 h at RT.

For quantitative analysis, six sections from each animal were arbitrarily selected (a total of 18 sections). The fluorescence images were observed and captured with a fluorescence microscope system (Axio Imager A2; Zeiss, Oberkochen, Germany). The number of positively stained small-diameter DRG neurons (i.e., <30 μm in diameter) (14) were counted, and the percentage of TRPV4-, IR-, or peripherin-positive neurons was calculated.

### Whole Cell Patch Clamp Preparation

#### DRG culture.

Rats were euthanized by bilateral thoracotomy, and the heart was removed under isoflurane anesthesia (4%–5% in 100% oxygen). The adequacy of anesthesia was verified by lack of a withdrawal response to tail pinch. DRGs were removed from all levels of the spinal cord of SD rats and digested with collagenase IV (1.0 mg/mL; Sigma-Aldrich, St. Louis, MO) for 30 min and trypsin-EDTA (0.05%; Sigma-Aldrich) for 5 min each at 37°C. Following this, the enzyme reaction was terminated by using trypsin inhibitor (0.08 mg/mL; Thermo Fisher Scientific) at RT. DRGs were then washed with Dulbecco’s modified Eagle’s medium (DMEM)/Ham’s F-12 (Thermo Fisher Scientific) supplemented with nerve growth factor (0.1 μg/mL, NGF-7S; Sigma-Aldrich), fetal bovine serum (10%; Sigma-Aldrich), Glutamax (1%; Thermo Fisher Scientific), glucose (0.8%; Sigma-Aldrich), penicillin–streptomycin (10 μL/mL; Sigma-Aldrich), and Dulbecco’s phosphate-buffered saline (Sigma-Aldrich). DRGs were placed on glass coverslips coated with poly-L-lysine (0.1 mg/mL; Sigma-Aldrich) and laminin (0.13%; Thermo Fisher Scientific) after being suspended and dissociated in the supplemented DMEM/Ham’s F-12 solution using a fire-polished Pasteur pipette. DRGs were maintained at 37°C in a CO_2_ incubator replacing with fresh supplemented medium at least every 2 days up to the day of current recording.

#### Whole cell patch clamp recording.

Inward current and membrane voltages from cultured small DRG neurons (i.e., <30 μm in diameter) were measured at RT as described previously (2, 7). Before each experiment, we observed cells using an inverted microscope (IX70; Olympus Corp, Tokyo, Japan) with a digital microscope camera (Olympus DP-25; Olympus Corp, Tokyo, Japan). Cell size was measured using open-source software, XnViewMP (XnSoft Corp, Reims, France). Inward currents were recorded in the voltage clamp mode at a holding potential of −90 mV. The patch pipettes were pulled from borosilicate glass capillaries (Narishige, Tokyo, Japan) and filled with a solution containing (mM): 122.5 K-gluconate, 12.5 KCl, 0.2 EGTA, 8 NaCl, 2 MgATP, 0.3 Na_3_GTP, and 10 HEPES. The pH was adjusted to 7.3 with KOH. The bath solution contains 119 NaCl, 2.5 KCl, 26 NaHCO_3_, 2.5 CaCl_2_, 1.3 MgSO_4_, and 25 glucose; the pH was 7.4. Based on an earlier study (15), patch and bath solutions were prepared.

We recorded currents and membrane voltages using Axopatch 200B (Molecular Devices, San Jose, CA). Data were digitized and analyzed using a Digidata 1550B (Molecular Devices) and Clampex software (Molecular Devices).

#### Mechanical stimulation and procedures in isolated DRG neurons.

Mechanically activated (MA) inward currents were recorded as described previously (4, 7). Mechanical stimuli were applied to the cell surface using a heat-polished glass pipette (φ = ~5μm) as a mechanical probe, which was positioned at an angle of ~45 degrees to the cell surface and driven by a piezo-controlled micromanipulator (Nanomotor MM3A; Kleindiek Nanotechnik, Kusterdingen, Germany). From the starting position where the mechanical probe gently touched the cell surface, the probe moved forward to press the cell surface and was kept at this position for 0.5 s and then moved backward to the starting position for each stimulation. The velocity of the movement was 1.8 μm·ms^−1^. A series of mechanical stimuli were applied, in which the intensity (distance of the probe movement) was increased from 1 to 6–12 steps (a step size was 1 μm/step; i.e., step number = the distance the probe moved in μm), with 10-s intervals. Based on the previous definition (16), the mechanical threshold was defined as the lowest intensity that evoked a MA current whose amplitude exceeded 50 pA. In this study, we compared both mechanical threshold and current amplitude for each test substance at the same step number corresponding to the intensity that mechanical threshold was marked before the application as a MA current amplitude.

The present study consisted of three application trials. One trial was applied to each cell, and MA currents were recorded before and after the application. In each trial, first, MA current was recorded to a stepwise-increasing mechanical stimuli up to 6–12 steps, as mentioned earlier. Then, using a pipette, the following test solutions were carefully and locally applied into the extracellular bath solution: *1*) HEPES buffered solution (control trial), *2*) 0.07% dimethyl sulfoxide (DMSO): 0.03% ethanol solution (vehicle trial), *3*) 5 U/mL insulin (Novolin R; Novo Nordisk Inc., Princeton, NJ) (insulin trials), or *4*) 5 U/mL insulin + 1 μM HC067047 trials (insulin + HC067047 trial). 1.0 mg HC067047 (a TRPV4 antagonist; Sigma-Aldrich) was dissolved in 2.12 mL of 70% DMSO: 30% ethanol solution to 1 mM. Then, 1 mM HC067047 stock solution was diluted with vehicle solution to 1 μM. The final concentrations of DMSO and ethanol were 0.07% and 0.03%, respectively. The concentration of HC067047 was based on the previous study (17). Five minutes after the application of the test solution, MA current was again recorded to the same series of mechanical stimuli at the original position.

Based on time constant (τ) of MA current inactivation, MA currents have been divided into three types: rapidly adapting (RA), intermediately adapting (IA), and slowly adapting (SA) (18, 19). We calculated the τ for current inactivity using a single exponential fitting procedure, via Clampfit software (Molecular Devices) and defined the currents as RA, τ < 3 ms; IA, 3 ≤ τ ≤ 30 ms; and SA, τ > 30 ms, according to previous studies (4, 7, 20).

#### Chemical stimulation and procedure.

About 10 μM GSK1016790A (a selective TRPV4 agonist; Sigma-Aldrich)-induced inward current was measured before and 5 min after the application of the following test solutions: *1*) HEPES-buffered solution as a vehicle control (control trial) or *2*) 5 U/mL of insulin (insulin trial). Those test solutions were locally applied using a pipette. We also pretreated DRG neurons with either *3*) 20 nM GSK1838705 (an IR antagonist; Sigma-Aldrich) (insulin + GSK1838705 trial), *4*) 500 nM wortmannin (a PI3K inhibitor; Sigma-Aldrich) (insulin + wortmannin trial), or *5*) 1 μM bisindolylmaleimide I (BIM)(a PKC inhibitor; Sigma-Aldrich) 2–4 h before the insulin trial (insulin + BIM). Five minutes after the application of test solutions, GSK1016790A-induced inward current to the same GSK1016790A stimulus was measured again. The concentration (10 μM) and exposure time of GSK1016790A (20~30 s) were identical before and after the applications.

About 1.0 mg GSK1016790A was dissolved in 1.53 mL of 70% DMSO: 30% ethanol solution to 1 mM. Then, the stock solution was diluted with bath solution to 10 μM. The final concentrations of DMSO and ethanol were 0.7% and 0.3%, respectively. GSK1838705 (1.0 mg) was dissolved in 1.88 mL of DMSO to 1 mM. Then, the stock solution was diluted with culture media to 20 nM. The final concentration of DMSO was 0.00002%. About 1.0 mg wortmannin was dissolved in 2.33 mL of 70% DMSO: 30% ethanol solution to 1 mM. Then, the stock solution was diluted with culture media to 500 nM. The final concentrations of DMSO and ethanol were 0.0035% and 0.0015%, respectively. BIM (1.0 mg) was dissolved in 2.24 mL of 70% DMSO: 30% ethanol solution to 1 mM. Then, the stock solution was diluted with culture media to 1 μM. The final concentrations of DMSO and ethanol were 0.07% and 0.03%, respectively. The concentrations of GSK1016790A (4), wortmannin (21), and BIM (22) were consistent with those used in earlier published studies.

Neurons were defined as GSK1016790A sensitive if the response to GSK1016790A was >50 pA. Total charge transfer was calculated by using Clampfit software (Molecular Devices) for comparison among the trials.

### Western Blotting

DRG neurons were isolated from all levels of the spinal cord of SD rats and digested with collagenase IV (10 mg/mL; Sigma-Aldrich) for 30 min and trypsin-EDTA (0.05%; Sigma-Aldrich) for 5 min each at 37°C. These ganglia were then dispersed by mechanically trituration with glass pipettes. The pellet from low-speed centrifugation was divided into two and resuspended in insulin-free culture media. The cells were plated on poly-l-lysine-coated culture dish and maintained at 37°C in an atmosphere of 95% CO_2_ for 24 h. Culture media were replaced with either fresh medium containing 5 U/mL insulin (100 μL insulin/2 mL culture media) or insulin-free culture media 5 min before sample collection. After incubations, the cultured DRG neurons were homogenized in icecold radioimmunoprecipitation assay lysis buffer (EMD Millipore, Temecula, CA) containing 50 mM Tris-HCl (pH 7.4), 150 mM NaCl, 0.25% deoxycholic acid, 1% NP-40, 1 mM ethylenediaminetetraacetic acid, protease inhibitor cocktail (Sigma-Aldrich), and PhosSTOP (Roche, Basel, Switzerland). The lysates were centrifuged at 700 *g* for 5 min at 4°C. Protein concentrations of the supernatants were measured with a bicinchoninic acid assay kit (Thermo Scientific).

Samples were prepared in Laemmli sample buffer with 5% 2-mercaptoethanol and heated 5 min in a heating block at 95°C. Equal amounts of sample proteins were subjected to sodium dodecyl sulfate-polyacrylamide gel electrophoresis with 10% resolving gels and then transferred to polyvinylidene difluoride membranes at 200 mA for 90 min. After transfer, membranes were blocked 5 min at RT in Bullet Blocking One (Nacalai Tesque, Inc., Kyoto, Japan). Membranes were incubated overnight with the following primary antibodies at concentrations of 1:1,000–10,000 at 4°C: anti-PI3K (1:1,000, Cat. No. 4292, RRID# AB_329869; Cell Signaling Technology, Danvers, MA), anti-phosphorylated (phospho)-PI3K (1:500, Cat. No. PA5-104853, RRID# AB_2816326; Invitrogen), anti-Akt (1:1,000, Cat. No. 9272, RRID# AB_329827; Cell Signaling Technology), anti-phospho-Akt (1:1,000, Cat. No. 9271, RRID# AB_329825; Cell Signaling Technology), anti-PKC (1:1,000, Cat. No. sc-8393, RRID# AB_628142; Santa Cruz Biotechnology), anti-phospho-PKC (1:1,000, Cat. No. ab23513, RRID# AB_2237450; Abcam), and anti-β-tubulin (1:10,000, Cat. No. ab131205, RRID# AB_11156121; Abcam).

After incubation with primary antibodies, membranes were rinsed and then incubated for 1 h at RT with secondary antibodies (Donkey anti-Mouse IgG, Cat. No. 715-035-150, RRID# AB_2340770 or Donkey anti-Rabbit IgG, Cat. No. 711-035-152, RRID# AB_10015282; Jackson ImmunoResearch Labs, West Grove, PA) diluted 1:5,000 in Can Get Signal Immunoreaction Enhancer Solution 2 (Toyobo, Osaka, Japan). Bands were visualized using a SignalBright Plus Chemiluminescent Substrate (Proteintech Group Inc., Rosemont, IL) and quantified using the Odyssey Fc Imaging system (LI-COR Biosciences, Lincoln, NE). WesternSure pen (LI-COR) was used to visualize the protein ladder. The intensities of immunobands were normalized to β-tubulin. We presented the total for protein contents as a fold expression relative to the mean values observed in HEPES group.

### Statistical Analysis

Data for patch clamp measurements to mechanical stimulation were analyzed by repeated two-way analysis of variance (ANOVA) and included main effects of test solution (HEPES, vehicle, insulin, or insulin + HC067047) and trial (before or after application of test solutions). The paired *t* test was used to compare the protein expression in Western blotting. One-way ANOVA was performed to compare parameters among the five trials in patch clamp measurements to chemical stimulation. When a significant interaction between test solution and trial or a significant difference among the trials was indicated, the Tukey’s multiple comparison test was used for post-hoc analyses.

Analyses were conducted using statistical software (Prism 9.0; GraphPad Software, San Diego, CA). Statistical significance was defined as *P* < 0.05. Data are presented as means ± SD.

## RESULTS

### Colocalization of TRPV4 and Insulin Receptor in Peripherin Positive Small DRG Neurons

**Figure 1.**
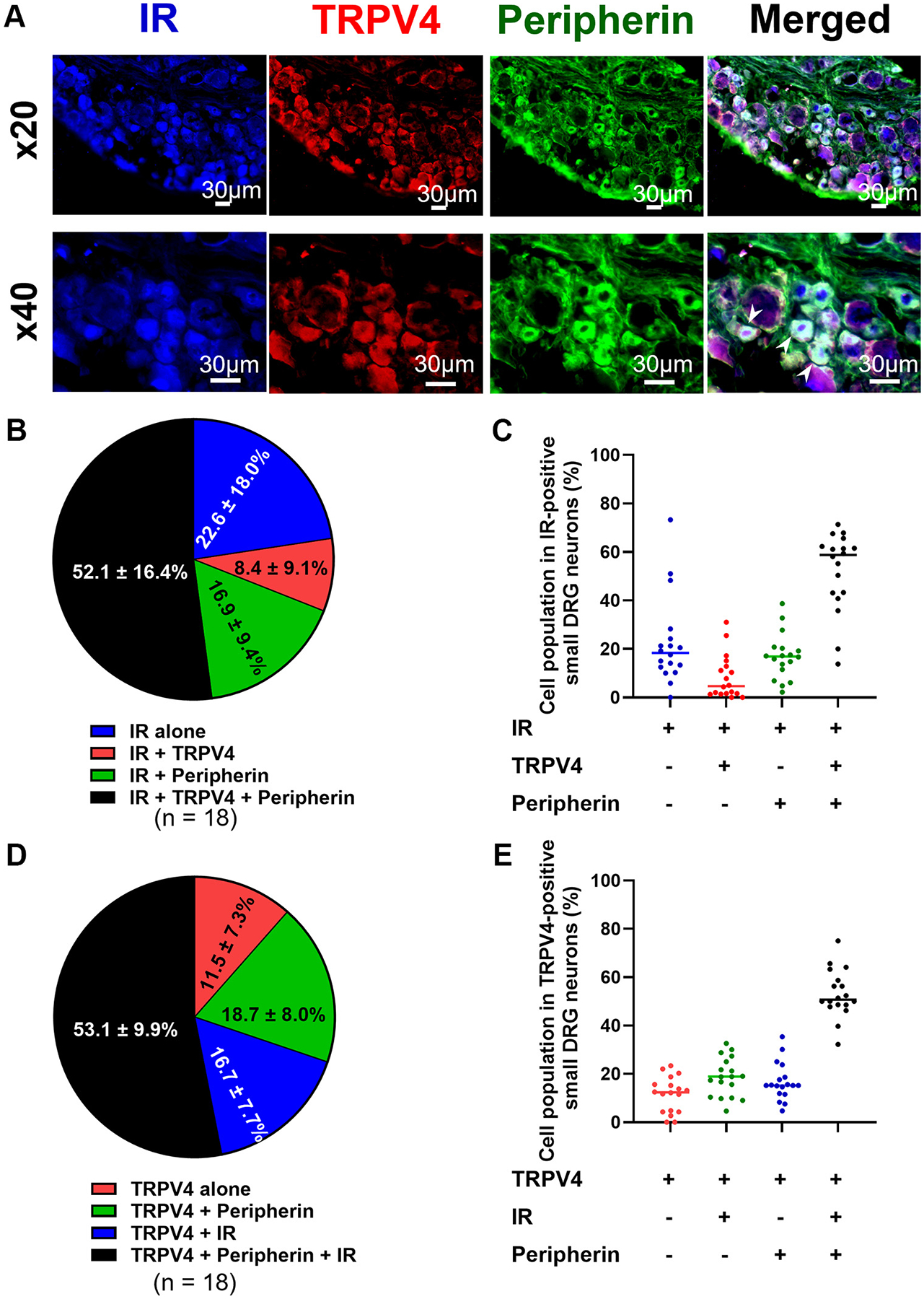
Colocalization of transient receptor potential vanilloid 4 (TRPV4) and insulin receptor (IR) in C-fibers of L4–L6 dorsal root ganglion (DRG) neurons in rats. *A*: representative images showing the expression of TRPV4 and IR-positive neurons in C-fibers of L4-L6 DRGs. Merged immunofluorescence images show colocalization of TRPV4, IR, and peripherin in small DRG neurons (white arrowhead). Magnifications: × 20 and ×40. Scale bar: 30 μm. The percentages of IR- and/or peripherin-positive neurons in TRPV4-positive small DRG neurons (*B*) and individual data obtained from distinct sections (*C*). The percentages of TRPV4- and/or peripherin-positive neurons in IR-positive small DRG neurons (*D*) and individual data obtained from distinct sections (*E*). Data are shown as means ± SD. A total of 18 sections were collected from 3 male Sprague-Dawley (SD) rats.

A total of 18 DRG sections were randomly collected from three male SD rats (BW: 314 ± 44 g, 12 wk of age). Figure 1A shows representative images of TRPV4 and IR-positive neurons in C-fiber L4-L6 DRGs. A total of 60.5 ± 25.4% of IR-positive small DRG neurons were colocalized with TRPV4 and 52.1 ± 16.4% of IR-positive neurons examined expressed TRPV4 and peripherin (Fig. 1, B and C).

About 69.8 ± 9.1% of TRPV4-positive small DRG neurons expressed IR. Of these, 53.1 ± 9.9% were peripherin-positive neurons (Fig. 1, D and E).

### Whole Cell Patch Clamp Recording of DRG Neurons

#### General results.

DRGs were collected from 15 male SD rats (BW: 310 ± 46 g, 7–12 wk of age). Small DRG neurons were used to assess the response to mechanical (*n* = 53) and chemical stimuli (*n* = 81) (φ = 26.6 ± 2.4 μm, range: 19.0–29.9 μm). The pipette resistance was 7.8 ± 1.6 MΩ, and the membrane potential was −49.4 ± 8.9 mV before mechanical or chemical stimulation. There were 28 MA currents classified as RA (53.8%), 20 as IA (37.7%), and 5 as SA (9.4%). The population of MA current type was significantly different among three trials (*P* = 0.0012, Fisher’s exact test).

#### Response to mechanical stimulation.

Mean mechanical threshold was 5.1 ± 1.8 steps (*n =* 53) (step number corresponded to the distance that the mechanical probe moved forward to push down the cell surface, i.e., μm) and the amplitude of MA current at the intensity corresponding to mechanical threshold was −95.3 ± 70.4 pA (*n =* 53).

Two, three, and two datasets were excluded from analysis for mechanical threshold in HEPES, insulin, and insulin + HC067067 trials, respectively, because the amplitude of MA currents did not exceed 50 pA at the maximal intensity, and mechanical threshold was not observed after the application of test solutions. However, these datasets were included to analyze the amplitude of MA current because we succeeded in recording changes at the intensity corresponding to mechanical threshold before the application of test solutions. In addition, two datasets were excluded from analysis for MA current amplitude in the insulin + HC067047 trial because we were not able to measure MA inward current at the same intensity that mechanical threshold was marked before the application of test solution. However, mechanical threshold was observed after the application of test solutions so that those datasets were included in the results of mechanical threshold.

**Figure 2.**
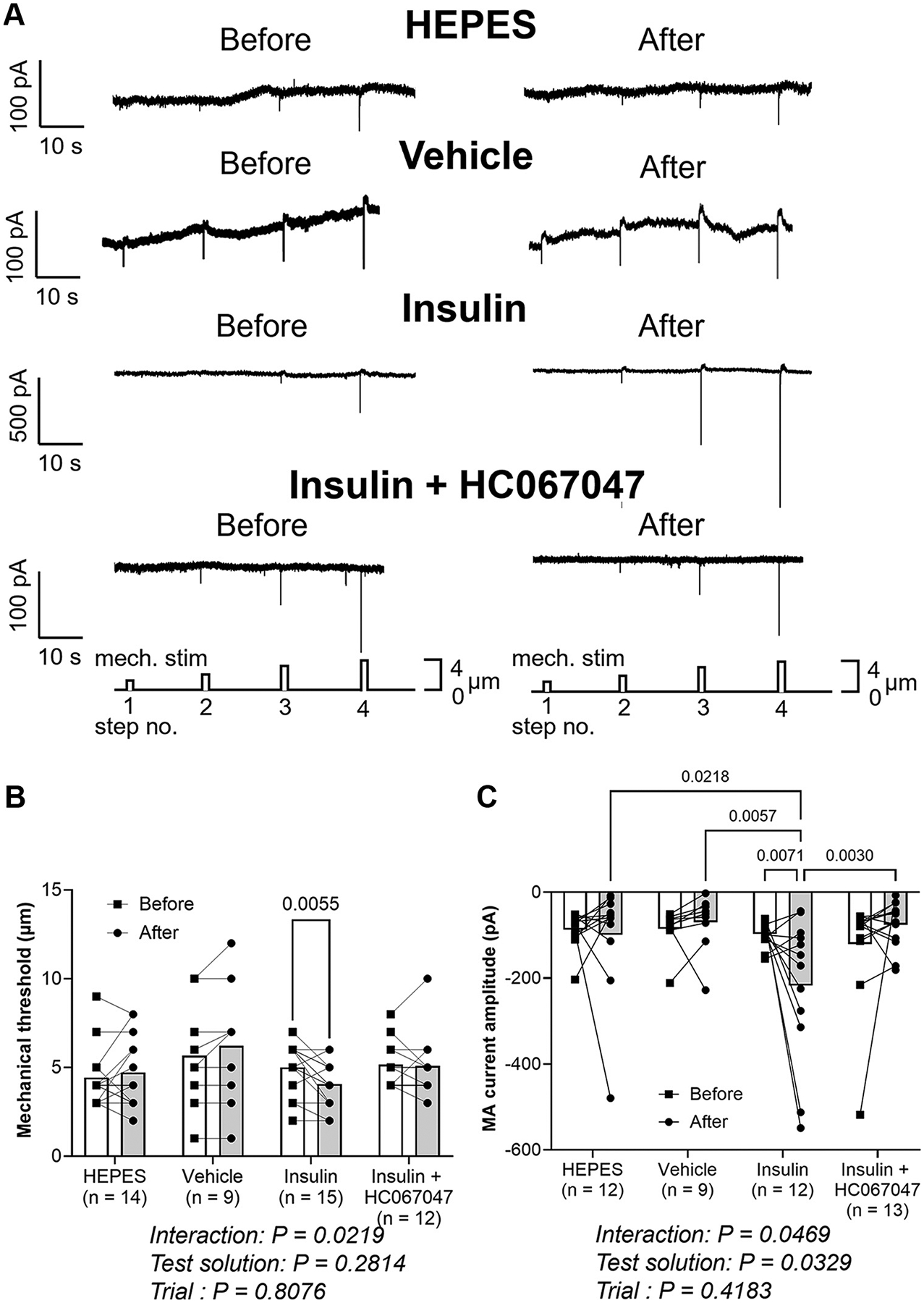
Sample recordings of mechanically activated (MA) inward currents in small DRG neurons before and 5 min after the application of HEPES-buffered solution (HEPES), 0.07% DMSO + 0.03% ethanol (vehicle), 5 U/mL insulin (insulin), or 5 U/mL insulin + 1 μM TRPV4 antagonist solution (insulin + HC067047) (*A*) and average changes in mechanical threshold (*B*) and in MA current amplitude (*C*) in small DRG neurons from before to 5 min after the application of test solutions. Data are shown as means ± SD. Two-way ANOVA was performed, followed by the Tukey comparison test. DRGs were collected from 15 male SD rats, and 53 small DRG neurons were assessed. ANOVA, analysis of variance; DRG, dorsal root ganglia; SD, Sprague-Dawley; TRPV4, transient receptor potential vanilloid 4.

Figure 2A shows sample recordings of MA currents before and 5 min after the application of either HEPES, vehicle, insulin or insulin + HC067047. A significant interaction between test solution (HEPES vs. vehicle vs. insulin vs. insulin + HC067047) and trial (before vs. after) was observed for mechanical threshold (*P* = 0.0219, Fig. 2B). A subsequent post hoc test revealed that mechanical threshold was significantly decreased 5 min after the application of insulin (*P* = 0.0055, Fig. 2B). Such insulin-induced reduction in mechanical threshold was not observed in either HEPES (*P* = 0.3937, Fig. 2B), vehicle (*P* = 0.1861, Fig. 2B), or insulin + HC067047 trials (*P* = 0.8172, Fig. 2B).

There was also a significant interaction between test solution and trial for MA current amplitude (*P* = 0.0469, Fig. 2C). MA current amplitude was significantly increased by insulin application (*P* = 0.0071, Fig. 2C) and that value was significantly higher than 5 min after the application of HEPES (*P* = 0.0218, Fig. 2C), vehicle (*P* = 0.0057, Fig. 2C), and insulin + HC067047 (*P* = 0.0030, Fig. 2C). MA current amplitude was not increased after the application of either HEPES (*P* = 0.7832, Fig. 2C), vehicle (*P* = 0.7513, Fig. 2C), or insulin + HC067047 (*P* = 0.2792, Fig. 2C).

**Figure 3.**
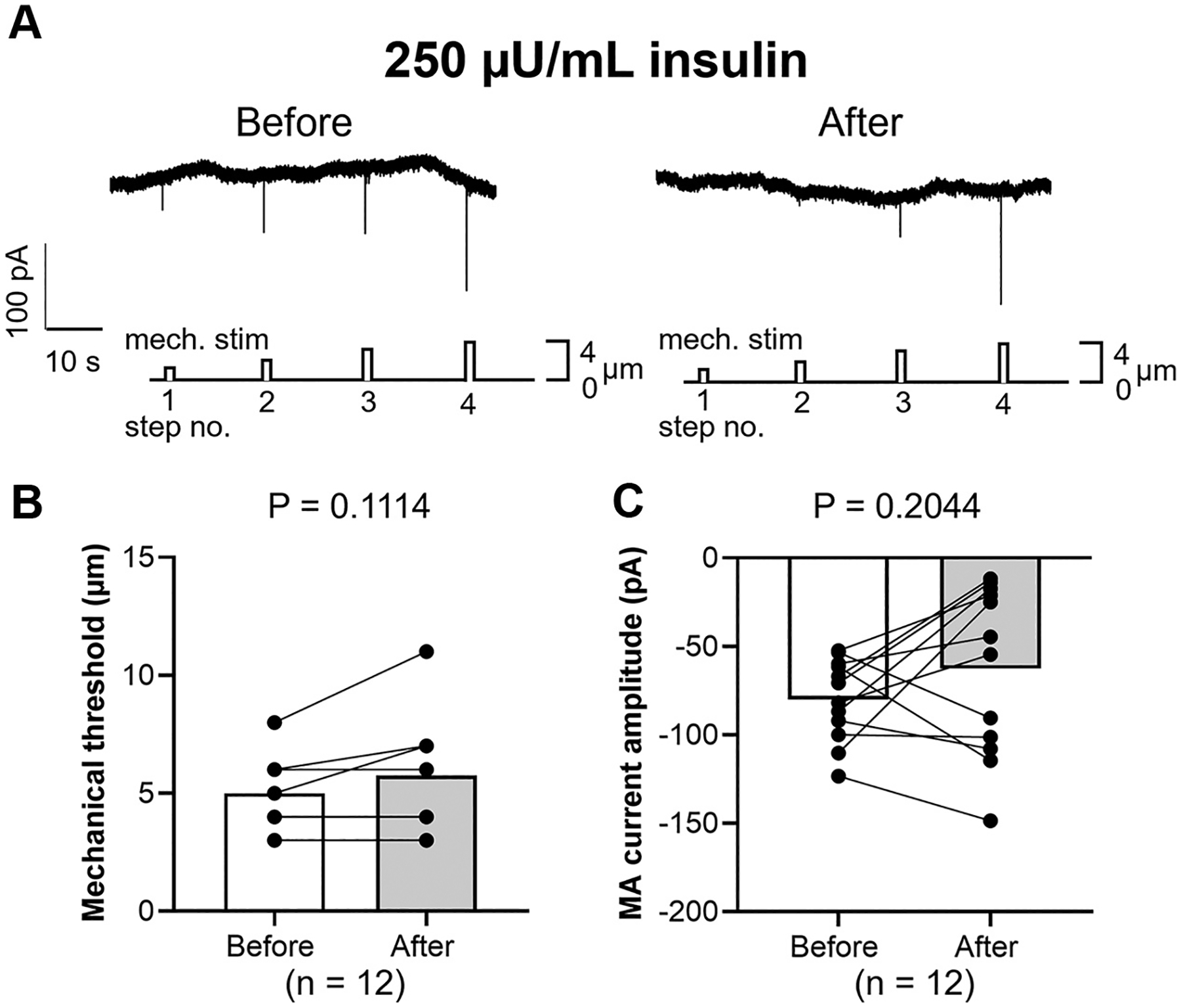
Sample recordings of mechanically activated (MA) inward currents in small DRG neurons before and 5 min after the application of 250 μU/mL insulin (*A*), average change in mechanical threshold (*B*), and average change in amplitude of MA current at the intensity corresponding to the mechanical threshold (*C*). Data are shown as means ± SD. Paired *t* test was performed. DRGs were collected from 3 male SD rats, and 12 small DRG neurons were assessed. DRG, dorsal root ganglia; SD, Sprague-Dawley.

In a corollary set of studies, mechanical threshold and MA current amplitude before and 5 min after the application of 250 μU/mL insulin were measured. Both mechanical threshold (*P* = 0.1114, Fig. 3B) and MA current amplitude (*P* = 0.2044, Fig. 3C) were not changed with 250 μU/mL insulin application.

### Western Blotting

**Figure 4.**
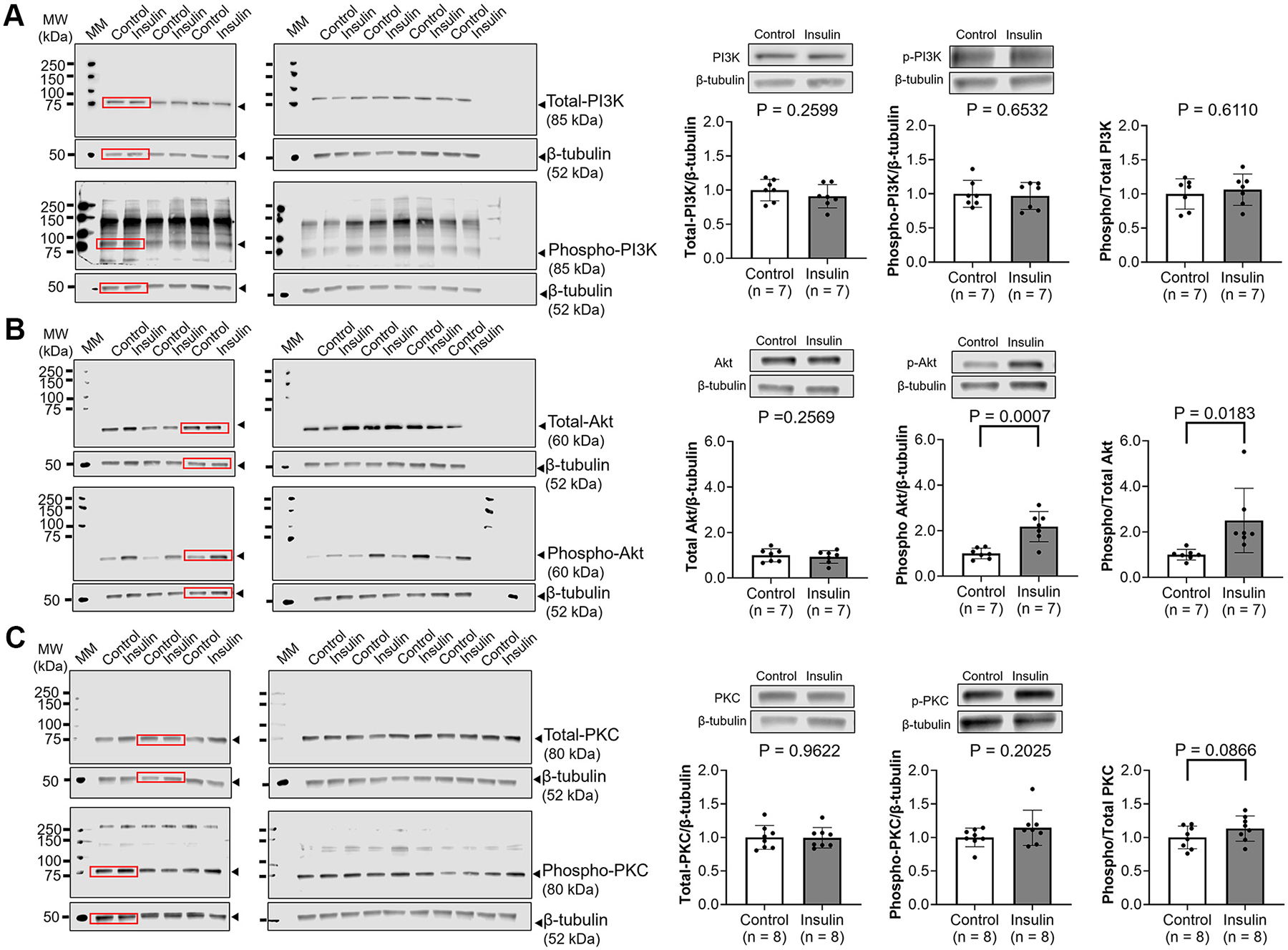
Western blot images of cultured DRG neurons incubated with/without 5 U/mL insulin. Protein expression of phosphoinositide 3-kinase (PI3K), phospho/total PI3K ratio (*A*), Akt, phospho/total Akt ratio (*B*), protein kinase C (PKC) and phospho/total PKC ratio (*C*) in cultured DRG neurons incubated with/without 5 U/mL insulin. WesternSure pen (LI-COR) was used to visualize the protein ladder. The representative bands are indicated by red rectangles in full blot images. Data are shown as means ± SD. Paired *t* test was performed. DRGs were collected from 7 (PI3K and Akt) and 8 male SD rats (PKC). DRG, dorsal root ganglia; MW, molecular weight; SD, Sprague-Dawley.

DRGs were collected from 7 (PI3K and Akt) to 8 male SD rats (PKC) (BW: 314 ± 37 g, 7–12 wk of age). Protein expression of total-PI3K (*P* = 0.2599, Fig. 4A), phospho-PI3K (*P* = 0.6532, Fig. 4A), total-Akt (*P* = 0.2569, Fig. 4B), total-PKC (*P* = 0.9622, Fig. 4C), phospho-PKC (*P* = 0.2025, Fig. 4C), and phospho/total PI3K ratio (*P* = 0.6110, Fig. 4A) in cultured DRG neurons was not different between control and insulin groups. Phospho/total PKC ratio in cultured DRG neurons incubated with insulin tended to be greater than that in control group, although statistical significance was not reached (*P* = 0.0866, Fig. 4C). Phospho-Akt (*P* = 0.0007, Fig. 4B) and phospho/total Akt ratio (*P* = 0.0183, Fig. 4B) in the insulin group were significantly higher than those in the control group.

### Response to Chemical Stimulation

**Figure 5.**
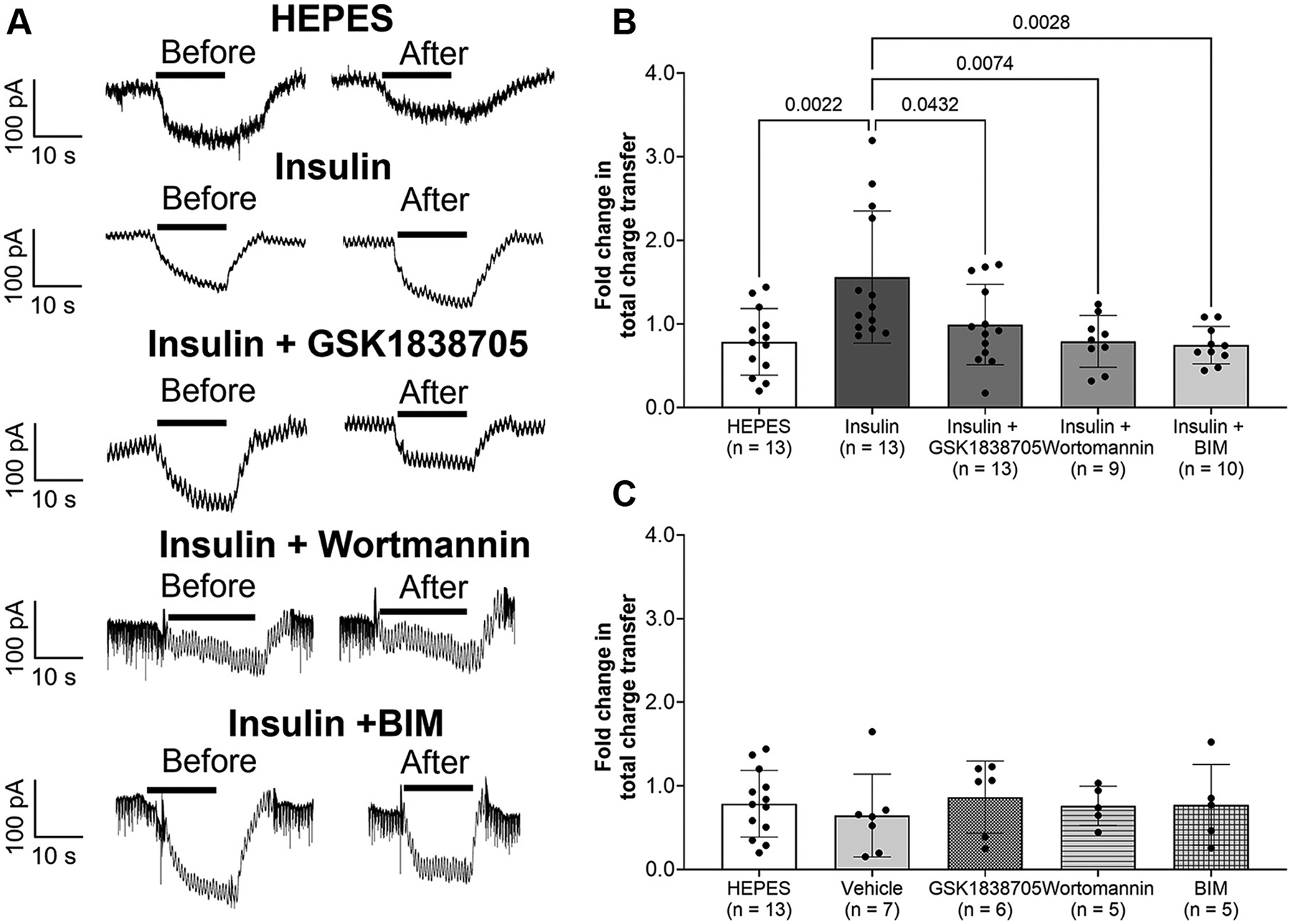
Sample recordings of 10 μM TRPV4 agonist (GSK1016790A) induced-inward currents in small DRG neurons before and 5 min after the application of HEPES-buffered solution (HEPES), 5 U/mL insulin (insulin), insulin after the pretreatment with 20 nM GSK1838705 (an insulin receptor inhibitor) (insulin + GSK1838705), insulin after the pretreatment with 500 nM wortmannin (a PI3K inhibitor) (insulin + wortmannin), or insulin after the pretreatment with 1 μM bisindolylmaleimide I (BIM)(a PKC inhibitor) (insulin + BIM) (*A*), fold change in total charge transfer in small DRG neurons from before to 5 min after the application of test solutions (*B*), and fold change in total charge transfer in small DRG neurons from before to 5 min after the application of HEPES-buffered solution (HEPES), 0.07% DMSO + 0.03% ethanol (vehicle), HEPES-buffered solution after the pretreatment with 20 nM GSK1838705 (GSK1838705), HEPES-buffered solution after the pretreatment with 500 nM wortmannin (wortmannin), or HEPES-buffered solution after the pretreatment with 1 μM BIM (*C*). Black bar indicates the period of exposure to 10 μM GSK1016790A solution. Data are shown as means ± SD. One-way ANOVA was performed, followed by the Tukey comparison test. DRGs were collected from 15 male SD rats, and 81 small DRG neurons were assessed. DRG, dorsal root ganglia; TRPV4, Transient receptor potential vanilloid 4; SD, Sprague-Dawley.

Sample recordings of GSK1016790A-induced inward currents before and 5 min after the application of test solutions are shown in Fig. 5A. Fold change in total charge transfer in the insulin trial was significantly greater than those in the other trials (vs. HEPES: *P* = 0.0022, vs. insulin + GSK1838750: *P* = 0.0432, vs. insulin + wortmannin: 0.0074, vs. insulin + BIM: *P* = 0.0028, Fig. 5B). No significant difference in fold change in total charge transfer was observed among the other trials. As a set of corollary experiments, we tested whether pre-incubation of inhibitors (GSK1838750, wortmannin and BIM) or vehicle (0.07% DMSO + 0.03% ethanol) itself alter GSK-1016790A-induced inward currents before and after the application of HEPES-buffered solution. There were no significant differences among the groups in fold change in total charge transfer (HEPES vs. vehicle: *P* = 0.9517, HEPES vs. GSK1838705: *P* = 0.9954, HEPES vs. wortmannin: *P* > 0.9999, HEPES vs. BIM: *P* > 0.9999, vehicle vs. GSK1838705: *P* = 0.8797, vehicle vs. wortmannin: *P* = 0.9895, vehicle vs. BIM: *P* = 0.9846, GSK1838705 vs. wortmannin: *P* = 0.9939, GSK1838705 vs. BIM: *P* = 0.9963, wortmannin vs. BIM: *P* > 0.9999, Fig. 5C).

## DISCUSSION

The major findings of this study were *1*) TRPV4 and IR were colocalized in C-fiber DRG neurons, *2*) insulin-induced mechanical sensitization in small DRG neurons was suppressed by blocking TRPV4 channels, *3*) the expression of proteins involved in the IR signaling pathway was enhanced in cultured DRG neurons incubated with insulin, and *4*) TRPV4 agonist-induced inward current was significantly augmented by insulin and such insulin-induced augmentation was significantly suppressed by both PI3K and PKC inhibitors.

Earlier studies have demonstrated that TRPV4 (9, 13, 23) and IR (12, 24) are highly expressed in DRG neurons of rats and mice. In the present investigation, we observed that 69.8% of TRPV4-positive small DRG neurons colocalized with IR (Fig. 1, D and E), suggesting that the colocalization of TRPV4 and IR may in fact be explained by the direct interaction of the proteins. In addition, 53.1% of TRPV4-positive small DRG neurons expressed both IR and the C-fiber marker peripherin (Fig. 1, D and E). Since C-fibers are polymodal in nature with many similarly responding to mechanical stimuli (25) and transmit peripheral signals to the central nervous system, thus processing important sensory and autonomic information (26), our results suggest that TRPV4 channels located in C-fiber DRG neurons may be associated with insulin-induced alteration of mechanosensation.

Our results in whole cell patch clamp experiments (Fig. 2, B and C) showing that the insulin-induced mechanical sensitization are consistent with our previous study (4). Furthermore, blocking TRPV4 channels suppressed the sensitization by insulin, suggesting that TRPV4 may be a molecular candidate for the mechanosensitive channel that is enhanced by insulin in small DRG neurons. In fact, 66.7% (10 out of 15 in mechanical threshold and 8 out of 12 in MA current amplitude) of small DRG neurons were sensitized by insulin in whole cell patch clamp measurements (Fig. 2, B and C). This rate is consistent with our immunofluorescence findings, demonstrating that 60.5% of IR-positive small DRG neurons expressed TRPV4 (Fig. 1, B and C).

To evaluate which proteins are related to the insulin-induced enhanced sensitization of TRPV4 channels, we evaluated protein levels of PI3K, Akt, and PKC using western blotting in cultured DRG neurons incubated with insulin. Insulin is known to activate Akt and PKC directly or in a PI3K-dependent manner (27–31). Moreover, TRPV4 sensitization has been attributed to increase phosphorylation through PKC, particularly at the Ser824 site (32, 33). In the present study, the level of Akt phosphorylation was significantly increased and the level of PKC phosphorylation likewise tended to be greater in cultured DRG neurons incubated with insulin (Fig. 4, B and C), suggesting that increased Akt and PKC may contribute to the insulin-induced mechanical sensitization by enhancing sensitization of TRPV4 channels. Furthermore, it has been reported that TRPV4 binds to PI3K, and these complexes are recruited to the plasma membrane upon stimulation by transforming growth factor-β, whose responsiveness is increased by insulin (34). Unexpectedly, in this study, insulin did not increase total PI3K, phospho-PI3K, and phosphor-/total PI3K ratio in the cultured DRG neurons (Fig. 4A). This was unexpected as an earlier study demonstrated that the peak response of insulin to PI3K is ~15 min after exposure in rat cardiomyocytes (35). Thus, although speculative in nature, the apparent dissociation of reactivity between PI3K and Akt/PKC may be due to differences in the peak response to insulin exposure on the expression of each protein.

Since the protein levels related to the IR signaling pathway were increased in cultured DRG neurons incubated with insulin (Fig. 4, B and C), we next evaluated the effect of blockade of components of the IR signaling pathway such as IR, PI3K, and PKC on TRPV4 agonist-induced inward currents using whole cell patch clamp measurement. Results demonstrated that the responsiveness of 69.2% (9 out of 13 in insulin trial) of TRPV4-positive small DRG neurons was potentiated by insulin in response to TRPV4 agonist (Fig. 5B). In addition, insulin-induced potentiation of TRPV4 channel sensitization was not observed in 69.2% (9 out of 13 in insulin + GSK 1838705 trial) of TRPV4-positive small DRG neurons pretreated with IR agonist (Fig. 5B). This rate supports our findings that 69.8% of TRPV4-positive small DRG neurons were colocalized with IR (Fig. 1, D and E). Moreover, pharmacological blockade of PI3K and PKC suppressed the insulin-induced sensitization to TRPV4 agonist in small DRG neurons (Fig. 5B). Wortmannin is known to be an inhibitor of PI3K, which is upstream of Akt and PKC. It has also been reported that wortmannin blocks insulin-induced phosphorylation of Akt in adipose tissue and adipocytes (36, 37), as well as activation of PKC in rat brain (38). Since blocking PI3K and PKC had inhibitory effects on insulin-induced TRPV4 sensitization (Fig. 5B), it is plausible that insulin enhances the sensitization of TRPV4 channels through the PI3K/Akt/PKC pathway.

### Limitations of This Study

In this study, we started with the use of a supraphysiological concentration of insulin based on our previous findings (2, 4). A normal fasting level of plasma insulin is between 5 and 15 μU/mL (39–42). However, high insulin responses to glucose load have been observed in patient with diabetes (100~10,000 μU/mL) (43, 44), hepatic steatosis (200 μU/mL) (45), and late pregnancy (300 μU/mL) (46, 47). In the present study, contrary to our expectations, insulin-induced mechanical sensitization of DRG neurons by exposure to a physiological concentration of insulin (250 μU/mL) was not observed (Fig. 3). This finding suggests that acute infusion of physiological dose of insulin may not alter TRPV4 channel function in DRG neurons. That being said, since people with type 2 diabetes (T2DM) often receive once- or twice-daily insulin injections (>100 U per day) to achieve glycemic control (48–51), an acute increase exceeding normal physiological levels in local insulin may occur with repetitive injections. Furthermore, since chronic hyperinsulinemia is observed in people with obesity and metabolic disorders (52, 53), further investigations are needed to evaluate whether prolonged exposure to a physiological concentration of insulin induces sensitization to mechanical stimulation in DRG neurons by enhancing sensitization of TRPV4 channels as observed in this study. In addition, only male rats were used in this study. As a result, the results reported may not directly extrapolate to female rats. Sex differences have been reported to exist in the regulation of physiological functions such as bone metabolism (54) and blood pressure control (55) by TRPV4. Future studies are required to address these issues.

In the current investigation, we did not identify to which tissues the DRG neurons innervated, as it was beyond the scope of our current investigation. However, it has been well known that deep nociceptors (e.g., muscle, bones, and joints) are less sensitive to noxious stimuli than cutaneous nociceptors (56). As such, the findings of this study may vary depending on the tissue of origin. Future studies using labeled DRG neurons in patch clamp experiments are needed to determine whether there is a tissue-specific effect. Furthermore, we did not label C fiber marker in our patch clamp studies. That being said, evidence suggests that the conduction velocity of the dorsal root fibers is related with DRG cell size, classifying these neurons into four main groups: Aα (30–55 m/s), Aβ (14–30 m/s), Aδ (2.2–8 m/s), and C (<1.4 m/s) (57). DRG soma with C fibers are of a more uniform size and are restricted to the small (<25 μm) and medium-sized (25–35 μm) cells within the ganglia (58). As the average size of DRG neurons was 26.6 ± 2.4 μm (range: 19.0–29.9 μm) in the present study, it is very likely that most of the DRG neurons that were tested can be classified as C fibers.

### Clinical Implications

Diabetes is a chronic disorder that results from early defects in IR function and progresses from hyperinsulinemia to beta cell failure. Peripheral hyperinsulinemia is a pathophysiological characteristic of type 2 diabetes (T2DM), especially in pre- or early stage patients (59). Diabetes leads to several complications including nephropathy, retinopathy, and neuropathy. Diabetic neuropathy affects the peripheral nervous system in at least 50% of patients with diabetes (60), and painful diabetic peripheral neuropathy is present in nearly a quarter of people with diabetes (61, 62). However, there is currently no satisfactory therapy for insulin-induced diabetic neuropathy (63). It has been reported that ob/ob mice, a mouse model of T2DM with hyperinsulinemia, had a significant decrease in their mechanical withdraw reflex threshold evaluated by von Fry testing (64, 65). To the best of our knowledge, there is only one study showing the preventative effects of the TRPV4 channel antagonist, HC067047, on established mechanical allodynia in diabetic rats induced by streptozotocin (66). The findings from this study might provide insights into the mechanisms underlying the pathogenesis of mechanical hyperalgesia in diabetes.

Treatment-induced neuropathy in diabetes is a neuropathic pain and/or autonomic neuropathy that develops in the setting of rapid improvements in glycemic control in patients with a long history of hyperglycemia (67). It occurs shortly after the initiation of intensive treatment with insulin in patients with T1DM and T2DM (68–70). The findings of the present study showing insulin-induced TRPV4 channel sensitization may provide further insights into the mechanisms underlying acute insulin treatment-induced neuropathy after intensive glycemic control.

Increasing evidence suggests that physical training improves glycemic control and decreases insulin resistance in T2DM (71–73). However, it is well known that the pressor response to exercise is exaggerated in T2DM (74–79). Since excessive blood pressure elevation can increase the risk for an adverse cardiovascular event (80, 81), exercise therapy for patients with T2DM is often limited. The exercise pressor reflex (EPR) is an important neural mechanism that contributes to the control of cardiovascular system during exercise. The mechanical and metabolic components of the EPR are known as the mechanoreflex and metaboreflex, respectively. Stimulation of mechanically and metabolically sensitive channels expressed by group III and group IV muscle afferents correspondingly activates the mechanoreflex and metaboreflex (82). Recent studies from our laboratory showed that the EPR (77, 83), the muscle mechanoreflex (84), and the muscle metaboreflex (78) are abnormally heightened in T2DM as well as T1DM rats. However, the mechanisms underlying the augmented responsiveness of this reflex in T1DM and T2DM remain unclear. Previously, we reported that TRPV1 sensitivity is enhanced in group IV muscle afferent fibers of rats with T2DM (77). In addition, we demonstrated that insulin potentiates the response to capsaicin, a TRPV1 agonist, in DRG neurons in vitro and muscle afferents ex vivo in normal healthy rodents (2). Yet, whether TRPV1 directly plays a pivotal role in mechanical sensation remains controversial. Our group demonstrated that the sensitivity of group IV skeletal muscle afferent fibers to mechanical stimuli is abnormally increased in T1DM (83) and T2DM (84). However, which mechanically sensitive channels contribute to the enhanced afferent sensitivity remains unclear. The findings of this study suggest that TRPV4 may be one of the mechanoreceptors potentiated by hyperinsulinemia in T2DM and such an augmentation of channel currents may induce abnormal EPR activity in T2DM. Nevertheless, these possibilities are based solely on findings from in vitro experiments. Extensive in vivo (unanesthetized decerebrate rat model) and ex vivo (e.g., single-fiber recordings from a muscle–nerve ex vivo preparation) studies will be required to determine whether TRPV4 contributes to the generation of insulin-induced potentiation of both EPR function and action potential responsiveness to mechanical stimulation in group III and group IV fibers. In addition, it is known that the magnitude of response to mechanical stimulation in group III muscle afferent is higher than that in group IV muscle afferents (85, 86). Furthermore, group IV afferent fibers are generally more metabolically sensitive and group III fibers are more mechanically sensitive (86, 87). Thus, whether the results of the current investigation apply similarly to group III fibers is unknown. Further investigation is needed to address these issues.

In conclusion, insulin-induced sensitization to both mechanical stimulation and TRPV4 agonist application was attenuated by blocking TRPV4 channels and PI3K/PKC in small DRG neurons, respectively. These findings suggest that the IR signaling pathway involving PI3K and PKC potentiates mechanical response in small DRG neurons by enhancing sensitization of TRPV4 channels.

## Data Availability

The data supporting the present findings are available from the corresponding author upon reasonable request.
